# Randomized Phase 3 LEAP-012 Study: Transarterial Chemoembolization With or Without Lenvatinib Plus Pembrolizumab for Intermediate-Stage Hepatocellular Carcinoma Not Amenable to Curative Treatment

**DOI:** 10.1007/s00270-021-03031-9

**Published:** 2022-02-04

**Authors:** Josep M. Llovet, Arndt Vogel, David C. Madoff, Richard S. Finn, Sadahisa Ogasawara, Zhenggang Ren, Kalgi Mody, Jerry J. Li, Abby B. Siegel, Leonid Dubrovsky, Masatoshi Kudo

**Affiliations:** 1 Mount Sinai Liver Cancer Program, Icahn School of Medicine at Mount Sinai, Tisch Cancer Institute, Icahn (East) Building, 11th Floor, Room 11-70A, 1425 Madison Ave, New York, NY 10029 USA; 2 Translational Research in Hepatic Oncology, IDIBAPS, Hospital Clinic Barcelona, University of Barcelona, Barcelona, Catalonia Spain; 3 Institució Catalana d’Estudis Avançats (ICREA), Barcelona, Spain; 4 Hannover Medical School, Hannover, Germany; 5 Yale School of Medicine and Yale Cancer Center/Smilow Cancer Hospital, New Haven, CT USA; 6 David Geffen School of Medicine at UCLA, Los Angeles, CA USA; 7 Graduate School of Medicine, Chiba University, Chiba, Japan; 8 Zhongshan Hospital Fudan University, Shanghai, China; 9 Eisai Inc, Woodcliff Lake, NJ USA; 10 Merck & Co., Inc, Kenilworth, NJ USA; 11 School of Medicine, Kindai University, Osaka, Japan

**Keywords:** Intermediate-stage hepatocellular carcinoma, Lenvatinib, Pembrolizumab, Transarterial chemoembolization

## Abstract

**Purpose:**

Transarterial chemoembolization (TACE) is the standard of care for patients with intermediate-stage hepatocellular carcinoma (HCC). Lenvatinib, a multikinase inhibitor, and pembrolizumab, a PD-1 inhibitor, have shown efficacy and tolerability in patients with HCC, and adding this combination to TACE may enhance clinical benefit.

**Protocol:**

LEAP-012 is a prospective, double-blind randomized phase 3 study. Adults with confirmed HCC localized to the liver without portal vein thrombosis and not amenable to curative treatment, ≥ 1 measurable tumor per Response Evaluation Criteria in Solid Tumors 1.1 (RECIST 1.1), Eastern Cooperative Oncology Group performance status 0 or 1, Child–Pugh class A and no previous systemic treatment for HCC are eligible. Patients will be randomly assigned to lenvatinib once daily plus pembrolizumab every 6 weeks plus TACE or placebos plus TACE. Dual primary endpoints are overall survival and progression-free survival per RECIST 1.1 by blinded independent central review (BICR). Secondary endpoints are progression-free survival, objective response rate, disease control rate, duration of response and time to progression per modified RECIST by BICR; objective response rate, disease control rate, duration of response and time to progression per RECIST 1.1 by BICR; and safety.

**Statistics:**

The planned sample size, 950 patients, was calculated to permit accumulation of sufficient overall survival events in 5 years to achieve 90% power for the overall survival primary endpoint.

**Discussion:**

LEAP-012 will evaluate the clinical benefit of adding lenvatinib plus pembrolizumab to TACE in patients with intermediate-stage HCC not amenable to curative treatment.

*ClinicalTrials.gov* NCT04246177.

## Introduction

Liver cancer is a leading cause of cancer-related mortality globally [1, 2]. The most common primary liver cancer is hepatocellular carcinoma (HCC), accounting for up to 90% of all cases [1, 3]. In patients with intermediate-stage HCC (Barcelona Clinic Liver Cancer stage B ﻿[BCLC]) characterized by asymptomatic localized HCC (i.e., no macrovascular disease) [4–8] TACE has remained the standard of care for more than 15 years and is associated with median survival of 25–30 months [3, 8, 9].

Although survival benefit of TACE alone has been demonstrated in patients with intermediate HCC, particularly in the 50% of whom achieve objective response [10–12], there is evidence that patients with high tumor burden or who do not respond to TACE do not derive clinical benefit from this procedure [13, 14]. Additionally, it is formally contraindicated in certain patients, e.g., patients with macrovascular invasion or liver failure [4, 6, 8]. Therefore, there is an urgent need for novel therapies to improve outcome in this heterogeneous patient population.

Angiogenesis plays a role in tumor growth, and angiogenic growth factors such as vascular endothelial growth factor (VEGF) and fibroblast growth factor (FGF) are elevated in patients with HCC [15–17]. Patients with HCC have significantly higher serum VEGF levels compared with healthy individuals, and elevated levels are associated with venous invasion and advanced disease [15]. Furthermore, an elevated serum VEGF level is associated with significantly worse overall and disease-free survival. Similarly, FGF signaling is implicated in development and progression of HCC [16, 17].

Lenvatinib is a potent multikinase inhibitor that selectively inhibits VEGF receptors 1–3, FGF receptors 1–4, platelet-derived growth factor receptor α, RET, and KIT and is approved in the first-line treatment setting for patients with advanced HCC [18]. In an open-label phase 3 study, lenvatinib demonstrated non-inferiority to sorafenib in overall survival (OS) and showed a safety profile consistent with previous studies [19]. The anti-tumor activity of lenvatinib is related to anti-proliferative effects and selective inhibition of FGF-signaling pathways, the latter being a key differentiating feature between lenvatinib and other multikinase inhibitors such as sorafenib [20].

Intact immune surveillance is an important mechanism against neoplastic growth [21]. Programmed cell death protein 1 (PD-1) and its ligand, PD-L1, play a role in the immune response and tumor immune evasion [22–25]. In patients with HCC, PD-L1 is prognostic of outcome, with high PD-L1 expression associated with significantly poorer prognosis than low PD-L1 expression [26]. Additionally, PD-1 level correlates with disease progression and is predictive of post-operative recurrence [23]. PD-L2, another ligand of PD-1, has been found to be overexpressed on tumor cells and is associated with poor clinical outcomes, especially in patients with HCC [27].

Pembrolizumab is a humanized monoclonal antibody against PD-1 and when combined with lenvatinib has shown promising antitumor activity and a manageable safety profile in patients with unresectable HCC not amenable to TACE in the phase 1b study KEYNOTE-524 study (Eisai Study 116; NCT03006926) [28]. Restoring antitumor immune activity and inhibiting angiogenesis may complement the locoregional necrosis achieved with TACE [8].

High-level evidence is required to support therapies indicated in medical guidelines for the treatment and management of HCC. Recommendations for clinical trial design and endpoints in HCC establish the importance of appropriate patient selection criteria (e.g., BCLC stage, Child–Pugh classification), stratification and randomization factors (e.g., Child–Pugh classification, α-fetoprotein level, geographical region, albumin-bilirubin [ALBI] grade and tumor burden) and trial endpoints (e.g., OS and progression-free survival [PFS]) [9]. Here, we describe the rationale and design for the prospective, double-blind, randomized phase 3 LEAP-012 study (NCT04246177), which is being conducted to investigate the efficacy and safety of lenvatinib plus pembrolizumab in combination with TACE compared with TACE alone in patients with intermediate-stage HCC not amenable to curative treatment.

## Materials and Methods

**Fig. 1:**
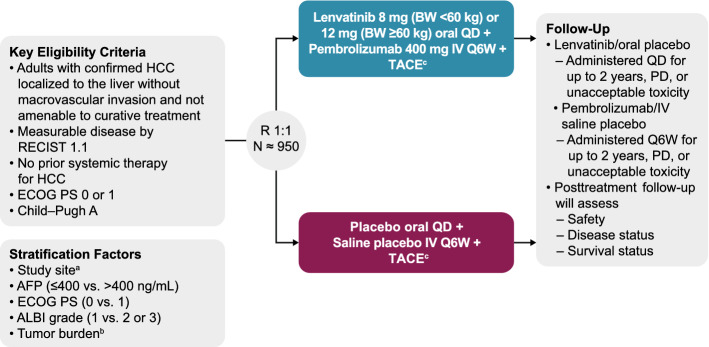
LEAP-012 study design. *AFP* α-fetoprotein; *ALBI* albumin-bilirubin; *BW* body weight; *cTACE* conventional TACE; *DEB*-*TACE* drug-eluting bead TACE; *ECOG PS* Eastern Cooperative Oncology Group performance status; *HCC* hepatocellular carcinoma; *IV* intravenously; *PD* progressive disease; *Q6W* once every 6 weeks; *QD* once daily; *R* randomization; *RECIST* Response Evaluation Criteria in Solid Tumors; *TACE* transarterial chemoembolization ^a^Stratification by study site was selected to minimize the effect of variations in TACE technique, instrumentation/imaging and other procedure-related heterogeneity across study sites. ^b^Tumor burden (6 and 12 rule): ≤ 6 vs. > 6 but ≤ 12 vs. > 12. Tumor burden = largest tumor size (in cm) + number of tumors. ^c^TACE will be limited to 2 treatments per tumors according to site-prespecified modality (cTACE or DEB-TACE)

LEAP-012 is designed to investigate oral lenvatinib plus intravenous pembrolizumab in combination with TACE compared with oral plus intravenous placebos in combination with TACE in patients with intermediate-stage HCC (Fig. 1). Patients will be randomly assigned 1:1 (stratified by study site, α-fetoprotein, Eastern Cooperative Oncology Group performance status, ALBI grade [29] and tumor burden) [30]. Stratification by study site was selected to minimize the effect of variations in TACE technique, instrumentation/imaging and other potential procedure-related heterogeneity across study sites. Specifically, each site is required to select the TACE modality (i.e., conventional TACE [cTACE] or drug-eluting bead–TACE [DEB-TACE]) and the chemotherapy agent (i.e., epirubicin, doxorubicin, or cisplatin) that will be used at that site. Other parameters, including chemotherapy dosing, DEB bead size, cTACE Lipiodol quantity, catheter size, and any additional agents used for hemostasis, will be selected by the interventional radiologist or hepatologist for each participant. Lenvatinib (or matching oral placebo) will be administered at 8 mg (< 60 kg) or 12 mg (≥ 60 kg) according to body weight orally once daily (QD) until disease progression or unacceptable toxicity; continuation of lenvatinib beyond 2 years of therapy requires consultation with the sponsor. Pembrolizumab (or matching saline placebo) will be administered at 400 mg intravenously every 6 weeks (Q6W) for up to 2 years or until disease progression or unacceptable toxicity. TACE will be administered per site prespecified modality (e.g., cTACE or DEB-TACE).

Systemic therapy with lenvatinib and pembrolizumab or matching placebos is planned to begin on day of random assignment (or, in special circumstances, up to 3 days after random assignment) and the first TACE will be administered 2 to 4 weeks after the start of systemic therapy. TACE is limited to 2 treatments per lesion, and the second treatment of any lesion is only permitted after confirmatory imaging per Response Evaluation Criteria in Solid Tumors 1.1 (RECIST 1.1) determines there is remaining viable tumor. If split TACE (a second procedure targeting previously untreated tumors) is required, it must be performed ≥ 1 month after the first TACE and before the first imaging evaluation. Lenvatinib will be held 2 days before and ≥ 7 days after TACE, with resumption contingent on postembolization syndrome recovery.

## Eligibility Criteria

Eligibility criteria are described in Table 1. Briefly, patients must be ≥ 18 years old with confirmed diagnosis of HCC by radiology according to American Association for the Study of Liver Diseases guidelines [5], histology or cytology (fibrolamellar and mixed hepatocellular/cholangiocarcinoma subtypes are not eligible) that is localized to the liver without portal vein thrombosis and not amenable to curative treatment.

**Table 1:** Eligibility criteria for LEAP-012

Key inclusion criteria	Key exclusion criteria
Age ≥ 18 years HCC confirmed by radiology, histology or cytology HCC localized to the liver without macrovascular invasion, confirmed by BICR, and not amenable to curative treatment ≥ 1 measurable HCC tumor based on RECIST 1.1, confirmed by BICR ECOG PS 0 or 1 Child–Pugh class A Amenable to TACE + chemotherapy agent prespecified at the study site: all tumors treatable with TACE Adequate organ function	Extrahepatic disease Eligible for liver transplantation HCC tumors measuring ≥ 10 cm in any dimension, > 10 HCC tumors confirmed by radiology, or HCC tumors occupying ≥ 50% of the liver volume, confirmed by BICR Esophageal or gastric variceal bleeding in the past 6 months; or clinically diagnosed hepatic encephalopathy in the past 6 months unresponsive to therapy; or uncontrolled, clinically apparent ascites Past systemic chemotherapy, including anti–VEGF therapy, or any systemic investigational anticancer agents for HCC Past therapy with an anti–PD-1, anti-PD-L1, or anti-PD-L2 agent or with an agent directed to another stimulatory or coinhibitory T-cell receptor (e.g., CTLA-4, OX-40, or CD137) Past locoregional therapy to existing liver lesions, including TACE, transarterial embolization, TARE, hepatic arterial infusion, or radiation for HCC. Past use of ablation and resection are permitted if > 4 weeks before first dose of study intervention. Past use of other locoregional therapy to lesions that have resolved is permitted if > 6 months before first dose of study intervention

*BICR* blinded independent central review; *CTLA-4* cytotoxic T-lymphocyte–associated protein 4; *ECOG PS* Eastern Cooperative Oncology Group performance status; *HCC* hepatocellular carcinoma; *PD-1* programmed death 1; *PD-L1* programmed death ligand 1; *PD-L2* programmed death ligand 2; *RECIST* 1.1 Response Evaluation Criteria in Solid Tumors, version 1.1; *TACE* transarterial chemoembolization; *TARE* transarterial radioembolization with yttrium-90; *VEGF* vascular endothelial growth factor

## Planned Sample Size and Study Period

The study sample size is ~ 950 and was calculated to permit the accumulation of sufficient OS events in 5 years to achieve 90% power for the OS primary endpoint. Patients will be randomly assigned in a 1:1 ratio to oral lenvatinib plus intravenous pembrolizumab in combination with TACE or oral plus intravenous placebos in combination with TACE.

Recruitment for the LEAP-012 study began in April 2020 and is ongoing at 165 sites in Australia, Brazil, Chile, China, Colombia, Denmark, France, Germany, Hungary, Ireland, Israel, Italy, Japan, Netherlands, New Zealand, Portugal, Puerto Rico, South Korea, Spain, Taiwan, Thailand, Turkey, Ukraine, UK and the USA.

## Outcomes and Endpoints

The dual primary endpoints of the LEAP-012 study are PFS assessed by blinded independent central review (BICR) per RECIST 1.1 and OS (Table 2). Secondary endpoints are PFS assessed by BICR per modified RECIST (mRECIST) [31]; objective response rate (ORR), disease control rate (DCR), duration of response (DOR) and time to progression (TTP), all assessed by BICR per RECIST 1.1 and mRECIST; and safety and tolerability. Tertiary/exploratory endpoints are PFS, PFS after the next line of therapy (PFS2), ORR, DCR, DOR and TTP (all assessed by the investigator per RECIST 1.1), biomarker analyses and patient-reported outcomes (PROs).

**Table 2:** Outcome measures and end points for LEAP-012

Dual primary endpoints	Definition
Progression-free survival assessed by BICR per RECIST 1.1	Progression-free survival is defined as time from randomization to the first documented disease progression or death due to any cause, whichever occurs first
Overall survival	Overall survival is defined as the time from randomization to death due to any cause

Secondary endpoints	Definition
Progression-free survival assessed by BICR per mRECIST	Previously defined
Objective response rate assessed by BICR per mRECIST	Objective response defined as complete response or partial response
Disease control rate assessed by BICR per mRECIST	Disease control is defined as a best overall response of complete response, partial response or stable disease Stable disease must be achieved at ≥ 6 weeks after randomization to be considered best overall response
Duration of response assessed by BICR per mRECIST	Duration of response is defined as the time from the first documented evidence of complete response or partial response until the first documented disease progression or death due to any cause, whichever occurs first
Time to progression assessed by BICR per mRECIST	Time to progression is defined as the time from randomization to the first documented disease progression
Safety and tolerability	Safety and tolerability assessments include: Adverse events, serious adverse events and hepatic adverse events Treatment discontinuations due to adverse events
Objective response rate assessed by BICR per RECIST 1.1	Previously defined
Disease control rate assessed by BICR per RECIST 1.1	Previously defined
Duration of response assessed by BICR per RECIST 1.1	Previously defined
Time to progression assessed by BICR per RECIST 1.1	Previously defined

Tertiary endpoints/exploratory outcomes	Definition
PFS assessed by the investigator per RECIST 1.1	Previously defined
Objective response rate assessed by the investigator per RECIST 1.1	Previously defined
Disease control rate assessed by the investigator per RECIST 1.1	Previously defined
Duration of response assessed by the investigator per RECIST 1.1	Previously defined
Time to progression assessed by the investigator per RECIST 1.1	Previously defined
Progression-free survival 2 assessed by the investigator per RECIST 1.1	Progression-free survival 2 is defined as the time from randomization to second/subsequent disease progression after initiation of new anticancer therapy including locoregional or systemic therapy, or death from any cause, whichever occurs first
Molecular (genomic, metabolic, and/or proteomic) biomarkers	Molecular (genomic, metabolic and/or proteomic) biomarker assessment includes determinants of response or resistance to treatments, using blood and/or tumor tissue
Health-related QOL EORTC QLQ-C30 EORTC QLQ-HCC18 EQ-5D-5L	Health-related QOL assessments include Global scores of the EORTC QLQ-C30 and EORTC QLQ-HCC18 Time to deterioration will be evaluated for EORTC QLQ-C30 and EORTC QLQ-HCC18 global health status/QOL Time to deterioration is the time to first onset of a 10 point or more decrease from baseline EQ-5D-5L health utility score

*BICR* blinded independent central review; *EORTC QLQ-C30* European Organisation for Research and Treatment of Cancer Questionnaire Core 30; *EORTC QLQ-HCC18* European Organisation for Research and Treatment of Cancer Quality of Life Questionnaire Hepatocellular Cancer; *EQ-5D-5L* EuroQol 5-dimension, 5-level questionnaire; *RECIST 1.1* Response Evaluation Criteria in Solid Tumors, version 1.1; *mRECIST* modified Response Evaluation Criteria in Solid Tumors; *QOL* quality of life

## Study Procedures

Tumor imaging will be performed by computed tomography or magnetic resonance imaging every 9 weeks until disease progression, the start of new anticancer treatment, withdrawal of consent or death, whichever occurs first. Objective response will be confirmed by a repeat imaging assessment performed at least 4 weeks after the first sign of complete or partial response. Following the first 9-week imaging scan, a second TACE may be performed to treat any previously treated tumors. TACE is limited to 2 treatments per tumor. In the case of treatment discontinuation without centrally verified disease progression, efforts to continue monitoring disease status by tumor imaging during treatment are encouraged.

Adverse events will be monitored throughout the study and up to 90 days (120 days for serious adverse events) after last dose or 30 days after last dose if the patient initiates new anticancer therapy, whichever occurs first during the follow-up period, and will be graded according to the Common Terminology Criteria for Adverse Events, version 5.0. PROs will be collected on day 1 of cycle 1 and every other cycle up to cycle 35.

## Statistics

Efficacy endpoints will be evaluated in the intention-to-treat population, which includes all randomly assigned patients analyzed according to randomized treatment group; DOR is based on the population of responders. The nonparametric Kaplan–Meier method will be used to estimate PFS and OS. The hypothesis of treatment difference in PFS and OS will be tested by a re-randomization test based on the stratified log-rank test, and a stratified Cox proportional hazards model with Efron’s method of tie handling will be used to estimate the magnitude of treatment difference. The stratified Miettinen and Nurminen method with weights proportional to the stratum size will be used for comparison of ORR between treatment arms. Safety analyses will be conducted in the as-treated population, which includes all randomly assigned patients who received ≥ 1 dose of study drug, according to the study intervention received. PRO analyses will be based on a PRO full analysis set population that includes patients who received ≥ 1 dose of study drug and completed at least 1 PRO assessment.

## Discussion

TACE has been the standard of care for intermediate-stage HCC for more than 15 years. Systemic therapy combined with TACE has not shown substantial improvements in efficacy. The LEAP-012 study will evaluate standard of care TACE in combination with lenvatinib plus pembrolizumab compared with TACE alone in patients with intermediate-stage HCC in a multicenter, double-blind, randomized phase 3 study.

## Declarations

## Conflicts of interest

JML has received personal fees for board membership from Bristol Myers Squibb and Celsion; for serving as a consultant to Bayer Pharmaceuticals, Eli Lilly, Bristol Myers Squibb, Eisai Inc. (Woodcliff Lake, NJ, USA), Celsion, Merck, Ipesen, Genetech, Roche, Glycotest, Nucleix, Sirtex, Mina Alpha Ltd. and AstraZeneca; participating in lectures/speaker bureaus for Bayer, Ipsen and Roche/Genentech; and the development of educational presentations from Medscape and Axis; and has received research grants payable to his institution from Bayer Pharmaceuticals, Bristol Myers Squibb, Eisai Inc. (Woodcliff Lake, NJ, USA), Ipsen and Boehringer Ingelheim. AV has received personal fees for serving as a consultant or honorarium from Merck Sharp & Dohme Corp., a subsidiary of Merck & Co., Inc., Kenilworth, NJ, and for serving as a consultant to Roche, Bristol Myers Squibb, Eli Lilly, Ipsen, Bayer, Pierre Fabre, AstraZeneca, Sanofi and Incyte. DCM has received personal fees for serving as a consultant or honorarium from Merck, Guerbert, Boston Scientific, Sirtex and Johnson and Johnson and for board membership from Quantum Surgical. RSF has received research grants payable to his institution from Merck, Bayer, Eli Lilly, Bristol Myers Squibb, Eisai Inc. (Woodcliff Lake, NJ, USA), Pfizer and Roche/Genentech. He has also received personal fees for serving as a consultant or honorarium and support for travel to meetings or for other purposes; participating in review activities such as data-monitoring boards, statistical analysis or endpoint committees; and provision of writing assistance, medicines, equipment or administrative support from Merck. He has also received personal fees for board membership from CS Stone and has received personal fees for serving as a consultant for Bayer, Eli Lilly, Bristol Myers Squibb, Eisai Inc. (Woodcliff Lake, NJ, USA), Pfizer, Roche/Genentech, AstraZeneca and Exelixis.

SO has received personal fees from Merck Sharp & Dohme Corp., a subsidiary of Merck & Co., Inc., Kenilworth, NJ, AstraZeneca and Chugai and personal fees and a grant from Bayer, Eisai Inc. (Woodcliff Lake, NJ, USA) and Eli Lilly. ZR has no conflicts of interest to disclose. KM is an employee of Eisai Inc. (Woodcliff Lake, NJ, USA) and has received personal fees for traveling to meetings from Eisai Inc. (Woodcliff Lake, NJ, USA). JJL is an employee of Merck Sharp & Dohme Corp., a subsidiary of Merck & Co., Inc., Kenilworth, NJ, USA. ABS is an employee of Merck Sharp & Dohme Corp., a subsidiary of Merck & Co., Inc., Kenilworth, NJ, USA. LD is an employee of Merck Sharp & Dohme Corp., a subsidiary of Merck & Co., Inc., Kenilworth, NJ, USA. MK has received personal fees for serving as a consultant for Eisai Inc. (Woodcliff Lake, NJ, USA); Ono; Merck Sharp & Dohme Corp., a subsidiary of Merck & Co., Inc., Kenilworth, NJ, USA; Bristol Myers Squibb and Roche and for lectures/speaker bureaus for Eisai Inc; (Woodcliff Lake, NJ, USA); Bayer, Merck Sharp & Dohme Corp., a subsidiary of Merck & Co., Inc., Kenilworth, NJ, USA; Bristol Myers Squibb and Eli Lilly, and research grants payable to his institution from Gilead Sciences, Taiho, Sumitomo Dainippon, Takeda, Otsuka, EA Pharma, AbbVie and Eisai Inc. (Woodcliff Lake, NJ, USA).
